# Circadian Functioning and Quality of Life in Substance Use Disorder Patients With and Without Comorbid Major Depressive Disorder

**DOI:** 10.3389/fpsyt.2021.750500

**Published:** 2021-10-29

**Authors:** Iman Hashemzadeh, Julia E. Marquez-Arrico, Kosar Hashemzadeh, José Francisco Navarro, Ana Adan

**Affiliations:** ^1^Department of Clinical Psychology and Psychobiology, School of Psychology, University of Barcelona, Barcelona, Spain; ^2^Department of Psychology, Fasa Branch, Islamic Azad University, Fasa, Iran; ^3^Department of Psychobiology, School of Psychology, University of Málaga, Málaga, Spain; ^4^Institute of Neurosciences, University of Barcelona, Barcelona, Spain

**Keywords:** substance use disorder, major depressive disorder, dual disorder, quality of life, circadian rhythm, sleep quality

## Abstract

**Aim:** Although a relationship between circadian disruption and development of several psychiatric disorders, such as major depressive disorder (MDD) and substance use disorder (SUD), has been observed, knowledge on this area is scarce yet. Therefore, this study aims to analyze the circadian functioning and quality of life (QOL) in SUD patients with and without comorbid MDD, two highly prevalent clinical entities with difficult therapeutic management.

**Methods:** One hundred sixty-three male patients under treatment, 81 with SUD and 82 with SUD comorbid major depressive disorder (SUD + MDD), were evaluated. For the circadian functioning assessment, we calculated Social Jet Lag (SJL) and used the reduced Morningness–Eveningness Questionnaire (rMEQ) and the Pittsburgh Sleep Quality Index (PSQI). QOL was measured using the shortened version of the World Health Organization's Quality of Life Questionnaire (WHOQOL-BREF). We collected sociodemographic and clinical variables to evaluate their possible influence on the circadian functioning. Intergroup differences among the variables were examined by different analyses of covariance (ANCOVA and MANCOVA). The possible relationships of quantitative clinical variables with rMEQ, PSQI, and WHOQOL-BREF were explored using bivariate correlation analysis.

**Results:** Lower SJL appears in the SUD + MDD group compared with SUD. The intermediate-type was more prevalent in the SUD group, while a higher percentage of morning-type patients was found in the SUD + MDD. Sleep quality (including latency and daytime dysfunction) was worse for SUD + MDD patients than for SUD even after controlling age and age of SUD onset variables. Last, QOL was poorer in patients with SUD + MDD and, for them, psychological health had a negative relationship with SJL and severity of depression.

**Conclusions:** Our data support and extend previous findings indicating that SUD + MDD is associated with worse clinical characteristics, more sleep problems, and poorer QOL than SUD patients. These results underline the importance of a precise assessment of these measurements in future studies conducted in SUD patients with/without MDD comorbidity that could be considered from a therapeutic point of view.

## Introduction

Most of the psychological and behavioral processes reveal circadian rhythms, with the sleep–wake cycle as one of their biological markers (1). In mammals, light is the pivotal environmental element that assists the biological clock to entrain to the environment (2). In the last century, with the initiation of using electrical lights and increased social demands, human sleep forms have been changed significantly. Social signals such as the timing of social interplays, mealtime, and exercise as non-photic synchronizers (3) can also influence our biological clock. Synchronization between endogenous circadian fluctuations (such as metabolic, physiological, or behavioral processes) and social synchronizers (such as zeitgebers and working hours) seems essential for physical and mental health, especially sleep (4, 5).

Circadian disruption deeply affects sleep, and it has been linked to the development of psychiatric disorders such as major depressive disorder (MDD) (3, 6, 7) and substance use disorder (SUD) (8, 9). The nature of human social interactions leads to the addition of social time to the circadian clock, which plays an important role in our vast daily habits (2). The relationship between this social time and the endogenous rhythms is a personal differential trait, known as chronotype or circadian typology (10). Based on circadian typology, individuals are categorized as morning type, intermediate type, and evening type. Morning types prefer to wake up early in the morning and find it hard to remain awake outside their usual bedtime, evening types desire to fall asleep late and have difficulty waking up in the morning, whereas intermediate types tend to do their tasks between morning- and evening-type times (10).

Most patients with mental disorders report the presence of sleep complaints, including insomnia, extreme daytime sleepiness, and early awakenings. These circadian dysfunctions may affect the daily functioning and quality of life (QOL) (7) of patients with recognized severe mental illness, such as depressive disorders (11, 12). In addition, sleep problems (SP) and circadian typology have been related to increased risk activities (13, 14) and onset of mental illness, especially depression (12, 15–17) and suicidality (18). Social jet lag (SJL) is an asynchrony between the biological clock of a person and the timing of social requirements such as faculty, school, or labor (19). It is a gap between internal circadian timing and typically early academic and/or work agendas or discrepancy between the weekend and weekday sleep timing (20, 21). Evening-type patients were found to be more vulnerable to SJL (1, 19, 22).

The term dual disorder refers to the coexistence or concurrence of at least one SUD and another mental disorder in the same person (23, 24). Dual disorder patients suffer higher rates of psychiatric hospitalizations (25), suicide attempts (26), more social problems and delinquent behaviors (24, 27), worse QOL (28–30), lower social consultation, and lesser use of social support (31), compared with those without comorbidity. A great body of research has indicated that substance use can exacerbate depression (27, 32, 33). The presence of an MDD increases the likelihood of developing an SUD and vice versa (34), although familial and genetic factors seem to be two risk factors for comorbidity of SUD and MDD (SUD + MDD) (35). Among SUD patients, the higher severity of depression was associated with both poorer self-rated health and QOL and more comorbidities than non-depressed ones (36). Patients with SUD + MDD presented worse prognosis and treatment outcomes than SUD patients (24, 31, 37). Moreover, more SUD dependence was reported in those with more comorbidities (38, 39). In addition, back pain, hypertension, and liver disease were the most frequently observed pathologies in SUD + MDD patients (36). So far, limited research has been done on SUD + MDD.

Circadian rhythmicity dysfunctions, especially SP, have a significant negative impact on physical and mental health, as well as on cognition and mood, and more importantly, they can be closely linked with substance use. The association between substance use and SP is mutual, but SP may establish a way for substance use and then self-remedies and make a vicious cycle in which each of these factors impairs each other (40). The relationship between SUD and evening type has been revealed in several studies; morning type seems to be a protection factor, while evening type is presently being considered as a risk factor for the beginning and progression of drug consumption (20, 37, 40). In this sense, regular sleep–wake habits may help to reduce addiction vulnerability and/or diminish the risk for worsening SUD (8).

Although the physiological and biological mechanisms common to depression and circadian rhythms are unclear (41), approximately 50–90% of MDD patients reported complaints about impairment of sleep quality (42). Recently, a study found that the average sleep regulatory index (measure that assesses the probability that an individual is awake) in persons without bipolar/depression disorder is higher than in those with such disorders (43). It has been suggested that MDD patients can recover from their depression by successfully handling their insomnia (44). It has been found that sanitation in sleep is related to amelioration of MDD symptoms (45) and SUD withdrawal (46). QOL and activities in the waking hours of individuals are greatly affected by their quality of sleep (7) and, reciprocally, daily stressors influence later bedtime, less sleep, and are associated with more mental health difficulties (47), so more research on this scope can be fruitful in the field of health and well-being. In comorbid disorders such as SUD + MDD, difficulties in social communication and request support (31) have been evidenced, an aspect that should be considered in the therapeutic management of these patients to improve both their recovery and their functionality. This work aims to draw further attention to several components of circadian functioning, which in turn should be considered as promising and worthy guidance for further non-pharmacological innovative therapies of SUD with and without depression. To our knowledge, this is the first study with this approach.

## Materials and Methods

### Participants

The total sample consists of 163 patients, all men, given the higher prevalence of these both considering the diagnosis of SUD and the patients under treatment. The sample collected included 81 SUD and 82 SUD + MDD patients who were under treatment at a psychiatric and an addiction center in Shiraz, Iran. The patients were selected according to their diagnoses following the fifth edition of Diagnostic and Statistical Manual of mental disorders (DSM-5) from the American Psychiatric Association (48). The inclusion criteria were (1) current SUD in remission for at least 1 month with dependence to several drugs (excluding alcohol because in Iran, it is illegal and people do not often use it), (2) participants between ages 18 and 55 years, (3) male gender, (4) abstinence period from 1 to 9 months with lack of SUD relapses for at least a month before participation, (5) current diagnosis of MDD in SUD + MDD patients, (6) being under treatment and with clinically stable psychiatric symptomatology, and (7) fluent in Persian language and native of Iran. The exclusion criteria were (1) having a current substance-induced psychiatric disorder or a psychiatric disorder due to a medical condition according to the DSM-5 criteria, (2) inability to complete instruments, and (3) receiving electroconvulsive therapy within 12 months before their study participation.

### Procedure

Each patient was informed of the study conditions by the clinical psychologist, prior to acceptance of participation. Measurement for all questions took 2 days and was conducted in two 2-h sessions for every patient and assessed individually in all cases. At first, the patients were divided into SUD, and SUD + MDD groups, based on the confirmatory diagnostic interview according to the DSM-5 criteria (2013). All patients were in treatment for their clinical conditions (SUD and SUD + MDD), with an integrated intervention in which addiction and mental health treatment are offered at the same time and by the same team. Integrated intervention includes a combination of motivational interviewing, contingency and case management, cognitive behavioral therapy, social skill training, and relapse prevention. This study was approved by the ethics committee of the Research Committee of the University of Barcelona (IRB00003099), and authorization from the research center of Shiraz University of Medical Science was also obtained.

### Measures

#### Structured Interview and Clinical Symptomatology Assessment

All selected patients were chosen by an experienced psychiatrist and two skilled clinical psychologists, individually, based on the Structured Clinical Interview (SCID-5-CV) of the DSM-5 (48). The participants were asked about sociodemographic (e.g., age, marital status, social class, education, economic status, and employment status) and clinical variables (e.g., diagnosis, age of SUD onset, type of drug use, abstinence time, daily use of medication, suicide attempts, presence of organic pathology, personal psychiatric history, labor, legal, and family problems), which were also extracted and reviewed from the medical history of centers. In addition, as clinical symptomatology assessment, SUD + MDD patients completed the Persian version of Hamilton Depression Rating Scale (HAMD-17) (49) to determine the severity of their MDD. The HAMD-17 total score ranges from 0 to 53, and it is interpreted as follows: 0 to 7, complete recovery; 8 to 13, mild depression; 4 to 18, moderate depression; 19–22, severe depression, and >23, severe depressive symptoms with adequate internal reliability for the present sample (0.793).

#### Circadian Functioning and Quality of Life Assessments

For the circadian functioning assessment, we calculated SJL and used the reduced Morningness–Eveningness Questionnaire (rMEQ) and the Pittsburgh Sleep Quality Index (PSQI). For SJL, we used a short four-item questionnaire that was evaluated by an interview and calculated as the differences between mid-sleep on workdays (MSW) and free days (MSF) (50, 51). The rMEQ is composed of five items and cut of the three circadian typologies as follows: 4–11 as evening type, 12–17 as intermediate type, and 18–25 as morning type. The scores range from 4 to 25 with higher scores indicating an inclination to morningness (52, 53) that in the present study showed an acceptable Cronbach's alpha coefficient for the total rMEQ (0.707). The PSQI evaluates seven dimensions of sleep: sleep quality, sleep latency, duration of sleep, efficiency of sleep, sleep disturbance, medication use, and daytime dysfunction. Each scale of the questionnaire takes a score from zero to three. On each scale, a score of 0 indicates a normal status, of 1, a mild problem, of 2, a moderate problem, and of 3, a severe problem and all scores together range from 0 to 21 (54) that in the present study showed an acceptable Cronbach's alpha coefficient for the total PSQI (0.710). Moreover, to determine the quality of life, we used the shortened version of the World Health Organization's Quality of Life Questionnaire (WHOQOL-BREF) that includes 26 items. Each item ranges from 1 to 5, in which higher scores indicated better QOL and encompassed four major domains (55) with achieved adequate Cronbach: physical health (0.730), psychological health (0.717), social relationships (0.746), and environment health (0.736). In addition, the WHOQOL-BREF includes two items that are related to the general QOL and to the general health status of the respondent (overall QOL), with an internal reliability of 0.743 and for the total WHOQOL-BREF of 0.906.

### Data Analysis

Descriptive statistics (i.e., mean, standard deviation, and frequencies) were calculated to describe the total sample and for each group. Group differences in demographic and clinical variables were explored with *t*-test or Mann–Whitney U-test for continuous variables and with Chi-square test for categorical variables. If the quantitative data fit the normal distribution, the *t*-test was used; otherwise, the non-parametric Mann–Whitney U-test was used instead. Internal consistence by Cronbach's alpha coefficient was calculated for the WHOQOL-BREF, rMEQ, and PSQI scales and dimensions. Intergroup differences for the total scores of the rMEQ and PSQI scales, considering the SUD and SUD + MDD, were examined by univariate analyses of covariance (ANCOVA). The group was considered as an independent variable and age as a covariate, since it could be a confounding factor. Also, a second ANCOVA analysis was performed adding the age SUD onset as the covariate. To study the differences of circadian typology in total PSQI, we performed ANCOVA analyses adding it as independent factor. Intergroup differences for the parameters of SJL and WHOQOL, considering the SUD and SUD + MDD groups, were examined by multivariate analyses of covariance (MANCOVA), where the group was considered an independent variable and age as a covariate. Besides, a second MANCOVA analysis was also performed adding the age of SUD onset as covariates. To investigate differences of circadian typology in WHOQOL and their dimensions, we performed MANCOVA analyses adding it as factor. The differences between levels of PSQI dimensions on WHOQOL-BREF dimensions were examined by MANCOVA tests, considering age as a covariate, and PSQI dimensions and group as independent factors. In all cases, *post-hoc* analyses were Bonferroni corrected, and partial Eta-square ($\eta_{\text{p}}^{2}$) was estimated to measure the effect size.

The relationships of quantitative clinical variables with rMEQ, PSQI dimensions, and WHOQOL dimensions were measured using bivariate correlation analysis. Then only significantly related variables were entered into the subsequent multiple linear stepwise regression analysis. The relationships between the total scores of QOL and PSQI were measured by Pearson correlation. All analyses were performed using the SPSS (Statistical Package for the Social Sciences version 26.0) software. The tests were two-tailed with the type I error set at 5%.

## Results

### Sociodemographic and Clinical Characteristics

According to Table 1, groups were not significantly different regarding age, marital status, socioeconomic, and employment status. The only difference in sociodemographic variables was observed for the academic studies, in which the SUD + MDD group reported more illiterate patients compared with SUD ones.

**Table 1:** Sociodemographic variables for the total sample, SUD, and SUD + MDD groups.

**Sociodemographic**	**Total sample**	**SUD**	**SUD + MDD**	**Statistical**
**data**		**(***N*** = 81)**	**(***N*** = 82)**	**contrast**
Age	39.29 ± 9.85	39.65 ± 10.19	38.94 ± 9.54	*t*_161_ = 0.463
Marital status				$\;\chi_{3}^{2}$ = 3.972
Single	19.6%	24.7%	14.6%	
Married	68.7%	61.7%	75.6%	
Divorced	8.0%	9.9%	6.1%	
Widow/widower	3.7%	3.7%	3.7%	
Socioeconomic status				$\;\chi_{3}^{2}$ = 5.409
High	16.6%	21%	12.2%	
Middle	44.2%	40.7%	47.6%	
Middle low	24.50	19.8%	29.3%	
Low	14.7%	18.5%	11%	
Academic class				$\;\chi_{4}^{2}$ = 9.740*
Illiterate	20.2%	13.6%	26.8%	
Primary studies	15.3%	13.6%	17.1%	
Middle school	14.7%	18.5%	11%	
High school	31.3%	32.1%	30.5%	
University studies	18.4%	22.2%	14.6%	
Employment status				$\;\chi_{2}^{2}$ = 2.755
Active	64.4%	60.5%	68.3%	
Inactive	28.8%	29.6%	28%	
Retired	6.7%	9.9%	3.7%	

*Mean, standard deviation, percentage, and statistical contrast (t-test or Chi-square test). SUD, substance use disorder; SUD + MDD, substance use disorder and comorbid major depressive disorder*.

* *p < 0.05*.

Concerning the studied clinical variables (Table 2), the *t*-test showed that there was no significant difference in the age of SUD onset between the two groups. In the concomitance of organic pathology, SUD + MDDs exhibited more medical disease comorbidities compared with the SUD ones (*p* = 0.012). Considering the personal psychiatric history, the SUD + MDD group showed a higher rate of previous history of mental disorders compared with the SUD group (*p* < 0.001). SUD + MDD patients showed more general anxiety and adjustment disorder history compared with the SUD ones (*p* = 0.012 and *p* < 0.001, respectively). Moreover, the groups also showed a significant difference in suicide attempts with higher rate for SUD + MDD patients (*p* = 0.013). In the field of law or legal problems, SUDs reported more conflicts compared with the SUD + MDD ones (*p* < 0.001). According to substance use, the SUD group reported a higher number of substance use (*p* = 0.009) and more opium, crystal, and heroin users compared with the SUD + MDD group (*p* < 0.030 in all cases). Moreover, groups were different in polydrug consumption, with more prevalence in the SUD patients than in the SUD + MDD group (*p* = 0.016). We did not find any significant difference in abstinence time between groups. Regarding the daily number of psychiatric medication, data revealed that groups were different; the SUD + MDD group used a higher rate of medication compared with the SUD ones (*p* < 0.001). According to the severity of depression of SUD + MDD (HAMD-17 scale), most of the patients reported moderate depression, although 46% of them are in the severe and very severe categories considered together.

**Table 2:** Clinical characteristics for SUD and SUD + MDD groups.

**Clinical data**	**SUD**	**SUD + MDD**	**Statistical**
	**(***N*** = 81)**	**(***N*** = 82)**	**contrast**
Age of SUD onset	35.80 9.22	36.25 ± 10.10	*t*_161_ = −0.299
Concomitance of organic pathology status^a^			$\;\chi_{6}^{2}$ = 12.910*
Total	33%	60%	$\chi_{1}^{2}$ = 6.368*
Hypertension	3.7%	1.2%	$\chi_{1}^{2}$ = 1.000
Hypothyroidism	2.5%	2.5%	$\chi_{1}^{2}$ = 2.778
Hyperthyroidism	3.7%	8.6%	$\chi_{1}^{2}$ = 0.200
Seizure	1.2%	8.6%	$\chi_{1}^{2}$ = 4.500*
Irritable bowel syndrome	1.2%	4.9%	$\chi_{1}^{2}$ = 1.800
Migraine	2.5%	6.2%	$\chi_{1}^{2}$ = 1.286
Other disorders(hepatitis B/C, Obesity, HIV, diabetes)	18%	28%	$\chi_{1}^{2}$ = 1.352
Personal psychiatric history^a^			$\;\chi_{6}^{2}\;=$ 18.813**
Relational Problems	6.2%	6.1%	$\chi_{1}^{2}$ = 0.000
General Anxiety Disorder	3.7%	15.8	$\chi_{1}^{2}$ = 6.250*
Panic disorder	6.2%	7.3%	$\chi_{1}^{2}$ = 0.091
OCD	4.9%	10.9%	$\chi_{1}^{2}$ = 1.923
Adjustment disorder	3.7%	43.9%	$\;\chi_{1}^{2}$ = 27.923***
Somatic disorders	4.9%	10.9%	$\chi_{1}^{2}$ = 1.923
Other disorders (eating disorders, PTSD, personality disorders)	13%	12%	$\chi_{1}^{2}$ = 0.048
Suicide attempts	0.26 ± 0.66	0.61 ± 0.95	*t*_161_ = 2.730**
Presence of problems			
Legal problems status	16%	0	${\;\chi }_{1}^{2}$ = 14.301***
Labor problem status	19.8%	9.8%	$\chi_{1}^{2}$ = 3.243
Family problems	28.4%	36.6%	$\chi_{1}^{2}$ = 1.246
Type of Substance^a^			
Nicotine	71.7%	57%	$\chi_{1}^{2}$ = 2.385
Opium	55.6%	39%	$\chi_{1}^{2}$ = 3.855*
Cristal	37%	17.1%	$\chi_{1}^{2}$ = 5.818*
Heroin	30.9%	13.4%	$\chi_{1}^{2}$ = 5.444*
Other (shire, hashish, Cannabis)	21%	32.9%	$\chi_{1}^{2}$ = 2.273
Number of substance use	2.04 ± 0.84	1.71 ± 0.75	*t*_161_ = 2.646**
Polydrug use	37%	17.2%	$\chi_{1}^{2}$ = 5.818*
Abstinence time (months)	7.50 ± 0.96	7.77 ± 1.47	*t*_143_ = 1.30
Daily use of psychiatric medication	31.43%	86.67%	$\chi_{1}^{2}$ = 21.253***
HAMD-17 scores		18.26 ± 5.36	
Without depression		2.4%	
Mild depression		12.2%	
Moderate depression		39%	
Severe depression		29.3%	
Very severe depression		17.1%	

*Mean, standard deviation, percentage, and statistical contrast (t-test or Chi-square tests). SUD, substance use disorder; SUD + MDD, substance use disorder and comorbid major depressive disorder; OCD, obsessive–compulsive disorder; PTSD, post-traumatic stress disorder; HAMD-17, Hamilton depression scale*.

a *Percentages will not equal to 100 as each patient may be in more than one category at the same time*;

* *p < 0.05*;

** *p < 0.01*;

*** *p < 0.001*.

### Circadian Functioning: Social Jet Lag, Circadian Typology, Quality and Components of Sleep

The differences among groups in bedtime during workdays (BTW) and bedtime during free days (BTF) were significant; the SUD group showed a delay in both BTW and BTF compared with the SUD + MDD ones (*p* = 0.006 in both cases). Concretely, the SUD went to bed later than the SUD + MDD patients during workdays and free days (weekend). Results were the same considering age and age of SUD onset as covariates (see Table 3).

**Table 3:** Social jet lag (SJL) and related parameters for the SUD and SUD + MDD.

			**Age as covariate**		**Age and age of SUD onset as covariates**	
	**SUD**	**SUD + MDD**				
	**(***N*** = 81)**	**(***N*** = 82)**	***F*** **_(1, 160)_**	** $\eta_{p}^{2}$ **	***F*** **_(1, 159)_**	** $\eta_{p}^{2}$ **
Bedtime during the workdays	00.22 ± 2.10	23.30 ± 1.49	7.787**	0.046	9.093**	0.054
Wake up during the workdays	7.43 ± 1.50	7.49 ± 1.55	1.521	0.009	1.774	0.011
Bedtime during the free days	00.45 ± 2.34	23.50 ± 1.58	7.763**	0.046	7.653**	0.046
Wake up during the free days	8.49 ± 2.06	9.26 ± 1.57	1.214	0.008	1.409	0.009
Social jet lag	1.35 ± 3.40	0.39 ± 0.75	3.350	0.021	3.897	0.024

*Mean scores, standard deviation with F and partial eta square ($\eta_{p}^{2}$(MANCOVA results)*.

*SUD, substance use disorder; SUD + MDD, substance use disorder and comorbid major depressive disorder*;

** *p < 0.01*.

According to circadian typology distribution (see Table 4), there is a significant difference between the studied groups ($x_{2}^{2}=8.777$). The SUD patients were more likely to be intermediate type in comparison with the SUD + MDD (*p* = 0.041), while the SUD + MDD patients were more prone to be morning type compared with the SUD group (*p* = 0.039). There was no bivariate correlation among rMEQ and the clinical variables.

**Table 4:** Results of the circadian typology for the total sample, SUD, and SUD + MDD patients.

	**Total sample**	**SUD**	**SUD + MDD**	**Statistical**
		**(***N*** = 81)**	**(***N*** = 82)**	**contrast**
Evening type	29.4%	27%	31%	$\;x_{1}^{2}\;$ = 0.333
Intermediate type	42.3%	53%	31%	$\;x_{1}^{2}\;$ = 4.188*
Morning type	28.2%	20%	36%	$\;x_{1}^{2}\;$ = 4.261*

*Percentages (Chi-square test)*.

*SUD, substance use disorder; SUD + MDD, substance use disorder and comorbid major depressive disorder;*

* *p < 0.05*.

The total score of rMEQ did not show any differences between the SUD (13.99 ± 3.98) and SUD + MDD (14.82 ± 4.51) groups in the ANCOVA analysis using age as a covariate $\left[F_{\left(1.160\right)}=1.607;\eta_{p}^{2}=0.010;p=0.207\right]$ and also by adding the age of SUD onset of the covariate $\left[F_{\left(1.159\right)}=1.621;\eta_{p}^{2}=0.010;p=0.205\right]$. Moreover, comparing the group means with the Iranian normative data (15.05 ± 3.71) (53) indicated that the SUD group presented lower scores than norms (*t*_347_ = 2.220, *p* = 0.014), and SUD + MDD provides data similar to the norm (*t*_348_ = 0.47, *p* = 0.321). In addition, analysis of the relationship with the clinical variables provided a significant difference between circadian typologies in polydrug consumers of the SUD group (*p* = 0.020), with polydrug more observable in the intermediate type compared with the morning type (*p* = 0.011) and evening type (*p* = 0.042).

The SUD group reported more frequency of “very good” quality of sleep in PSQI compared with the SUD + MDD group (*p* = 0.018), while the SUD + MDD group reported more frequency of “fairly bad” sleep quality in comparison with SUD (*p* = 0.011). Considering the sleep latency, the SUDs reported the minimum time compared with the SUD + MDD group (*p* = 0.001). In contrast, the groups did not differ in the total duration of sleep, sleep efficiency, and sleep disturbance. Use of medication to sleep was different between the two groups, and the SUD patients reported the lowest consumption of psychiatric drugs to fall asleep compared with the SUD + MDD patients (*p* = 0.013). Moreover, the SUD group had lower daytime dysfunction compared with SUD + MDD patients (*p* = 0.014) (see Table 5).

**Table 5:** Quality and dimensions of sleep for the SUD and SUD + MDD patients.

**Pittsburgh sleep quality index (PSQI)**	**SUD**	**SUD + MDD**	**Statistical**
	**(***N*** = 81)**	**(***N*** = 82)**	**contrast**
Sleep quality			$\;x_{3}^{2}\;$ = 12.389**
Very good	17.2%	4.8%	
Fairly good	53%	47.5%	
Fairly bad	17.2%	37.8%	
Very bad	12.3%	9.7%	
Sleep latency			$\;x_{3}^{2}\;$ = 20.660***
Lower than 15 min	56.8%	23.2%	
16–30 min	22.2%	41.5%	
31–60 min	17.3%	23.2%	
More than 60 min	3.7%	12.2%	
Sleep duration			$\;x_{3}^{2}\;$ = 2.390
More than 7 h	9.9%	14.6%	
6–7 h	34.6%	24.4%	
5–6 h	44.4%	48.8%	
Less than 5 h	12.2%	11.1%	
Sleep efficiency			$\;x_{3}^{2}\;$ = 4.853
More than 85%	21%	9.8%	
75–84%	4.9%	9.8%	
65–74%	24.7%	27%	
Lower than 65%	49.4%	53.7%	
Sleep disturbance			$\;x_{3}^{2}\;$ = 5.096
Not during past month	5%	0	
Less than once a week	74.1%	76.8%	
Once or twice a week	21%	22%	
Three or more times a week	0	1.2%	
Sleep medication			$\;x_{3}^{2}\;$ = 29.368***
Not during past month	43.2%	20.7%	
Less than once a week	18.5%	3.7%	
Once or twice a week	9.9%	6.1%	
Three or more times a week	28.4%	69.5%	
Daytime dysfunction			$\;x_{3}^{2}\;$ = 8.744*
No problem at all	32.1%	13.4%	
Only a very slight problem	25.9%	39%	
Somewhat of a problem	32.1%	35.4%	
A very big problem	9.9%	12.2%	

*Frequency with percentage and statistical contrast (Chi-square test)*.

*SUD, substance use disorder; SUD + MDD, substance use disorder and comorbid major depressive disorder;*

* *p < 0.05*;

** *p < 0.01*;

*** *p < 0.001*.

The total score of PSQI showed differences between groups [*F*_(1.160)_ = 25.661; $\eta_{p}^{2}=0.138;p<0.001$]; SUD + MDD patients reached the highest scores (11.56 ± 3.13), considered worst, compared with the SUD ones (9.12 ± 2.96). The second analysis considering age and age of SUD onset as covariates corroborated the same result $\left[F_{\left(1.159\right)}=24.708;\eta_{p}^{2}=0.134;p=0.001\right]$. Regarding the clinical variables associated with the total PSQI score, only the age of SUD onset (negatively) and HAMD-17 score (positively) were related in the SUD–MDD group, explaining 5.5% of its variance [F_(2, 79)_ = 4.672; *p* = 0.012]. Supplementary analysis for the total score of PSQI considering age as a confounding factor, and groups and circadian typology as factors indicated no interaction between them [*F*_(2.156)_ = 1.766; $\eta_{p}^{2}=0.022;p=0.174$]. Therefore, the main effect of circadian typology showed a significant difference in the total PSQI score $\left[F_{\left(2.156\right)}=3.938;\eta_{p}^{2}=0.048;p=0.021\right]$, with worse scores reported by evening types (10.60 ± 3.18) compared with intermediate types (9.34 ± 2.77) but no difference with morning types (10.48 ± 3.07) (*p* = 0.006 and *p* = 0.098, respectively).

### Quality of Life

**Figure 1:**
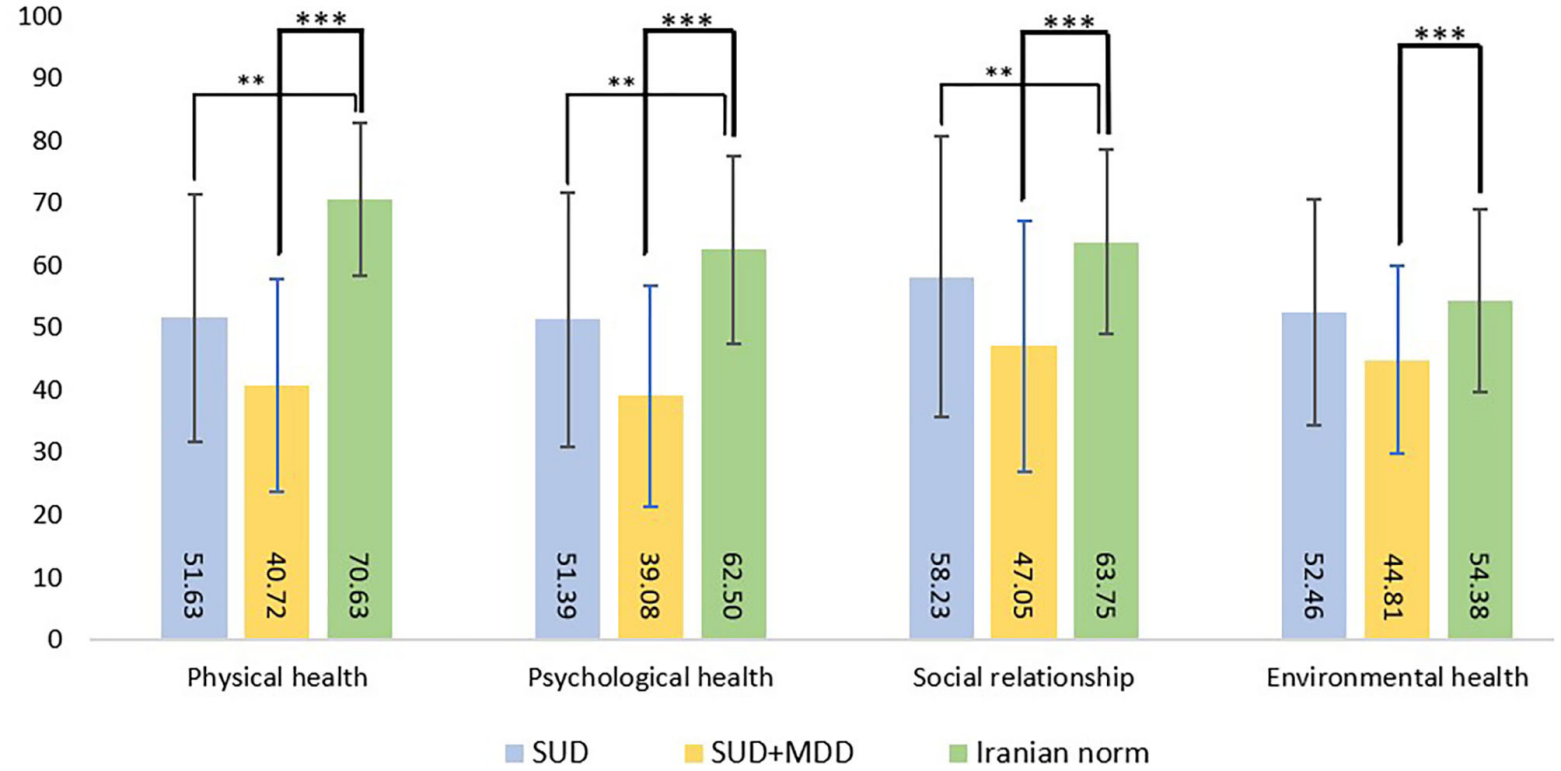
Mean differences and standard deviations for the dimensions of quality of life in the two groups of patients according to Iranian population norms. SUD, substance use disorder; SUD + MDD, substance use disorder and comorbid major depressive disorder. **p < 0.01; ***p < 0.001.

In comparison with Iranian population norms (55), the mean scores for all dimensions were lower in the SUD + MDD group of patients (*t*_986_ > 5.660, *p* < 0.001). Moreover, the subscales of physical health, psychological health, and social relationship of the SUD group scored lower than norms (*t*_986_> 3.050, *p* < 0.010 in all cases), while only environmental health was similar to the norm (*t*_986_ = 1.110, *p* = 0.134) (see Figure 1).

MANCOVA analysis using age as a covariate showed differences between groups in the WHOQOL-BREF scores and its dimensions (see Table 6). For the overall QOL, SUD patients presented the highest scores compared with SUD + MDD patients both considering age as covariate and age and onset of SUD as covariates. Results for all dimensions (physical health, psychological health, social relationship, and environmental health) also showed that SUD patients presented the highest scores compared with SUD + MDD.

**Table 6:** Quality of life results in the groups of SUD and SUD + MDD patients.

			**Age as covariate**		**Age and age of SUD onset as covariates**	
**WHOQOL-BREF**	**SUD**	**SUD + MDD**				
	**(***N*** = 81)**	**(***N*** = 82)**	***F*** **_(1, 160)_**	** $\eta_{p}^{2}$ **	***F*** **_(1, 159)_**	** $\eta_{p}^{2}$ **
Overall QOL	51.70 ± 24.10	44.05 ± 19.86	5.123*	0.031	6.219*	0.038
Physical health	51.63 ± 19.85	40.72 ± 17.08	15.062***	0.086	16.680***	0.095
Psychological health	51.39 ± 20.38	39.08 ± 17.80	17.220***	0.097	18.993***	0.107
Social relationship	58.23 ± 22.61	47.05 ± 20.00	11.087***	0.065	12.935***	0.075
Environmental health	52.46 ± 18.11	44.81 ± 15.06	8.790**	0.052	9.261**	0.055

*Mean scores, standard deviation, F and partial eta square ($\eta_{p}^{2}$) tests (MANCOVA results)*.

*WHOQOL-BREF, shortened version of the World Health Organization's Quality of Life Questionnaire; SUD, substance use disorder; SUD + MDD, Substance use disorder and comorbid major depressive disorder*;

* *p < 0.05*;

** *p < 0.01*;

*** *p < 0.001*.

Multiple regression analysis of WHOQOL-BREF and its dimension scales related to clinical variables indicated that within the SUD + MDD group, the SJL, explaining 4.1% of its variance, was negatively related to physical health [F_(1, 80)_ = 4.440; *p* = 0.038] and HAMD-17 scoring, explaining 6.5% of its variance was negatively linked to Psychological health [F_(1, 80)_ = 6.675; *p* = 0.012]. No variables were associated with overall QOL, social relationship, or environmental health in any group.

We used MANCOVA analysis to establish the differences between levels of PSQI dimensions on QOL dimensions. This analysis showed no interaction between sleep quality and groups in physical health, psychological health, and environmental health. Therefore, the main effect of sleep quality appeared significant in physical health [F_(3, 154)__=_ 6.947; $\eta_{p}^{2}=0.119;\;$ *p* < 0.001], that is, the patients who reported very good sleep quality had higher physical health (52.14 ± 18.47) compared with very bad ones (35.12 ± 17.12). Also, there was a significant difference between sleep quality levels in psychological health [F_(3, 154)__=_ 6.961; $\eta_{p}^{2}=0.119;\;$*p* < 0.001]. The patients who reported very good sleep quality had higher psychological health (51.11 ± 19.63) compared with very bad ones (36.67 ± 18.36). Moreover, a significant difference was observed between sleep quality levels in environmental health [F_(3, 154)__=_ 4.205; $\eta_{p}^{2}=0.076;\;$ *p* = 0.007]; thus, the patients who reported very good sleep quality had higher environmental health (52.29 ± 20.37) compared with very bad ones (38.96 ± 15.39). There was no evidence of significant difference in social relationship dimension.

MANCOVA analyses showed an interaction between sleep latency and physical health [F_(3, 154)_ = 4.582; $\eta_{p}^{2}=0.082;\;$ *p* = 0.004]. The significant difference was observed in the physical health of the SUD + MDD group among sleep latency levels (*p* = 0.011); thus, the patients who reported more than 60 min of sleep latency reported lower physical health (13.60 ± 3.10) compared with those with lower than 15 min of sleep latency (18.68 ± 5.61). In addition, a significant interaction was observed between sleep latency and psychological health [F_(3, 154)_ = 3.159; $\eta_{p}^{2}=0.058;\;$ *p* = 0.026]; the significant difference was observed in the psychological health of the SUD group among sleep latency levels (*p* = 0.024), that is, the patients with more than 60 mins of sleep latency reported lower psychological health (15.55 ± 3.90) compared with the other ones with lower than 15 min of sleep latency (18.80 ± 54.91). The correlation between scores of QOL and PSQI using the Pearson test also revealed a negative correlation between them (ρ = −0.186, *p* = 0.017). In the total sample, these data are suggesting that patients who reported better quality of sleep had a better QOL. The correlation performed for each clinical group did not provide differences in any of them. Besides, MANCOVA analysis adding circadian typology as a factor revealed no significant difference for this factor nor for their interaction with the group in the QOL scale.

## Discussion

The current study sought to explore the characteristics and possible differences of sociodemographic and clinical state in SUD and SUD + MDD Iranian patients under treatment, as well as their circadian functioning (SJL, circadian typology, quality of sleep) and QOL. We also aimed to compare data from our sample in rMEQ and QOL with population norms and identify clinical correlates of sleep and circadian rhythmicity characteristics of each diagnosis group.

Regarding the sociodemographic characteristics of the patients, in contrast with other studies, no differences were observed in marital (26, 30), employment, and socioeconomic status between groups (28), but a lower educational level in the SUD + MDD group (38, 39) was reported compared with the SUD ones. The reasons that can differentiate these results are the type of gender population as well as the conducted geographical areas and the age of SUD onset. In the present study, it can be concluded that SUD patients show a greater tendency to continue their education due to less mental and psychological conflicts than SUD + MDD patients. In other words, patients with two diagnoses like SUD + MDD are clinically suffering more severe illness than patients with only one disorder who also endure lower social support consultation (26, 31, 56). All this can be an obstacle to academic development, adding difficulties to the cognitive impairment that is part of the MDD diagnosis itself (57). From another point of view, cultural differences in each society, such as differences in financial and social levels and personality traits, can lead to different results in sociodemographic variables. Regarding more law and legal problems observed in the SUD group, these can be related to greater impulsivity (13, 58), lower disability pension, and lack of economic income (30).

Consistent with previous findings, we did not find any differences between groups in the age of SUD onset between groups (30). The SUD + MDD patients showed higher rates of medical disorder comorbidity (30, 37, 59) and more suicide attempts (26, 28, 30, 32, 37, 59, 60). In the current research, the SUD + MDD patients reported more personal psychiatric history than the SUD ones (39, 61, 62), which is in line with a recent study on SUD + MDD patients with lower mental health component compared with the SUD ones (30). These findings indicate that dual disorder patients are more prone to withdraw treatment and its follow up sessions (23, 59), which is associated with an increased risk of relapse and recurrence (63). Many dual patients turn to use substances as self-medication (64) trying to reduce either the severity of their illness or side effects of medication. This highlights the importance of designing and using more depth interventions in the future, focused on secondary psychiatric symptoms to achieve better adherence and therapeutic outcomes in SUD patients and especially for those with SUD + MDD.

In terms of substance use, our results indicated that in Iran, opium use is one of the consumption priorities among substances (65, 66), and crystal use is the second substance in the SUD group (67). As there is little known about the prevalence of SUD and severe mental illness comorbidity in Iran, future research should consider more precisely the related substances for the SUD + MDD patients. In contrast to Iranian investigations, European ones reported more nicotine, alcohol, cocaine, and cannabis use (26, 32, 68, 69) in dual patients without considering, in several cases, nicotine as a substance of abuse. Contrary to previous studies (30, 31), the SUD group reported more poly drug use and number of substance use compared with the SUD + MDD ones. The differences in the type of substance use vary according to the cultural and social structure of each society (70, 71), and conflicting results also could be attributed to the differences in any sample selection or availability of substance or environmental pressure to abuse substances. Therefore, the opium consumption in Iran can be due to its geographical proximity to Afghanistan, which is the largest producer in the world (66), while in countries far from the opium distribution route, consumption is minimal. Consistent with previous studies, the SUD + MDD group reported a higher rate of medication compared with the SUD ones (26, 30, 37, 59), in concordance with the therapeutic management of the dual diagnosis.

Lower SJL appears in the SUD + MDD group compared with the SUD one. These results are consistent with previous works that showed SUD is a predictor of both more SJL and sleep problems (20, 72, 73). Although these studies did not include patients with dual disorders, it seems that substance use, regardless of the comorbid mental disorders, can impact sleep times in clinical populations. Therefore, a strategy to regulate the levels of daily activity in the social–work schedules (40, 50) of SUD patients with and without comorbid depression is suggested.

We observed that in the SUD patients, the intermediate type was more prevalent, although it was lower in relation to population norms (53). By contrast, a study with SUD patients under treatment showed that the predisposition was toward the morning type (74), whereas other studies indicated that the SUD patients were more prone to the evening type (20, 75, 76). Differing results may be due to the fact that many previous studies have not considered a comorbid group or they compared the results with healthy participants. Moreover, some other suggested factors may be influencing the differences in circadian typology of SUD patients, including methodological aspects (only males or both sexes), the type of treatment (ambulatory vs. residential), as well as the abstinence time that if it is longer, it shows better rhythmic organization with a morning-type pattern. In our study, patients with SUD + MDD scored almost similar to the normal population (53), but they showed a higher percentage of morning-type compared with SUD in line with a previous study (77). Most studies reported that MDD patients are more prone to be of the evening type (17, 78) or intermediate type (79). These studies have not considered dual individuals, and most of them carried out evaluations coinciding with the diagnosis and compared the single mental disorders with normal population or healthy participants. So, in our case, the greater morningness could be explained by the treatment effect for SUD and also for MDD (37, 77, 78), since different treatment approaches agree that having a balanced and structured daily routine has a positive clinical effect (44). Although the physiological and biological mechanism links between circadian rhythms and mental disorders are unclear (37, 41), our results suggest the possibility of adjusting the circadian typology during treatment and that it should not be considered as a predictive factor in clinical features (80, 81).

Since previous findings have indicated that evening-type individuals had more polydrug use (20, 82), our result may extend such association also to patients with SUD and intermediate type suggesting that polydrug use may not be so specifically linked to evening typology. Therefore, future research may focus on the main type of substance of dependence, taking also into account the circadian typology as well as the cultural factors of the community. Although other studies on SUD patients showed that SJL and sleep irregular schedules are more related to eveningness (73), and to the presence of severity of depression (17), we did not find any association among SJL, circadian typology, and depression severity in SUD + MDD patients. A possible explanation of these non-observed differences may be the sleep schedule routines that the treatment for SUD introduces and the limitation of social life during recovery.

As far as we know, this is the first study that considers the quality of sleep in the SUD + MDD group in comparison with SUD in Iran. As we expected, the SUD + MDD patients showed the worse scores in PSQI and sleep quality, sleep latency, and daytime dysfunction dimensions, even after controlling age and age of SUD onset variables compared with patients with SUD. These results are in line with previous studies on the depressed patients, where it is observed that those who reported worse sleep quality have more severe depression (15–17, 47). We found this observation in the comorbid depressive group (SUD + MDD) in which the loss of sleep quality can be exacerbated due to the presence of SUD. A possible explanatory factor is that patients with SUD + MDD tend to report the highest use of sleep medication (28, 37), and they also may be suffering from more emotional problems (31) that can impact their sleep times in comparison with the SUD ones. We observed in the SUD + MDD group that the score of PSQI had a positive relationship with severity of depression, in concordance with studies in MDD patients (15, 78), and a negative relationship with the age of SUD onset. SP and MDD are associated with lifestyles, such as low light exposure, reduced physical movement (15), and overeating (83), and the unstable social rhythms also play a role in sleep and circadian dysfunctions in MDD (3, 6). This may harm the biological clock functioning and mood of individuals and increases the possibility of developing an MDD (12, 84). Therefore, the same can happen to SUD + MDD patients. In line with results on MDD patients (4, 42), we did not find any interaction among circadian typology and sleep quality in SUD and SUD + MDD patients. Probably, the quality of sleep plays a more important role than the circadian typology in the prediction of SUD, in agreement with evidence that SPs are important pioneers of substance use (85).

This study is one of the few made on the QOL of SUD + MDD patients in comparison with SUD, confirming the worst QQL in the dual group (28–30). SUD patients scored better in the overall QOL and all dimensions (physical, psychological, environmental health, and social relationship), even after controlling confounding factors like age and age of SUD onset. Having better physical health and social relationships in SUD patients than SUD + MDD is in line with a previous work, which also observed less functional disability and more work activities and social network (28). One study during the 12-week trial of treatment for cannabis users found a significant association between reduction of consumption and improvement in anxiety and depression of the patients, but they did not see any progress in their QOL (86). Also, in a recent study, relapses were observed related to the general health of the SUD + MDD group and physical functioning for the SUD patients, indicating the need to emphasize one or the other during the treatment depending on the diagnosis (30). Therefore, patients with SUD and, especially, with comorbidity, may have a more stressful life and subsequently worse QOL than the non-clinical population (26, 31, 59), which could be negatively associated with treatment fulfillment and prevention of recurrence.

We found in the SUD + MDD patients that better psychological health was related to a lower SJL and severity of MDD. This is consistent with previous studies on healthy participants in which lower SJL is related with minor depressive symptoms (87) and with SUD + MDD patients reporting lower mental psychological scores than depressive patients without SUD (58). Therefore, it seems that SJL has an important effect on both healthy and clinical population, which may exacerbate MDD even in SUD patients. Also, in the urban population, using devices such as smartphones and manipulating alarm clocks (88) has caused a deeper misalignment between social demands and the biological clock. As a result, these factors strongly affect the life of a person, especially physical and psychological functioning of patients with mental disorders. Since adequate sleep is important for health and the timing of sleep is necessary for social demands (13), more investigation is needed to consider the possible pathways that may influence and be influenced by SJL, social demands, and their interactions with the biological clock, especially in the clinical population (regarding more SP) compared with healthy individuals.

Our data are consistent with previous works, where MDD patients with a better QOL reported a higher quality of sleep (89–91). We extend these data and emphasize that patients with more physical, psychological, and environmental health show higher sleep quality. Moreover, both SUD + MDD patients, with more physical health, and SUD patients, with more psychological health, reported less sleep latency. This result is in line with a study in which a significant negative correlation among depression, QOL, and sleep quality was found (15), but are in contrast with another study (92). Summarizing, the role of SUD + MDD has not been adequately analyzed, especially in the QOL aspect, as it seems that any psychological and physical activity improvement in people with mental disorder (93–95) is contingent on improving sleep parameters (27, 93). Although further research is needed in this area, according to the results of previous research, it seems that sleep problems and related factors lead to exacerbating difficulties in both SUD and SUD + MDD populations and may underlie as precipitating factors in their development.

To enhance QOL of the patients and creating a protective factor against relapses after the treatment sessions, patients are recommended to follow healthy habits at appropriate times (40, 74). These included regular time patterns of meals, daily physical and social activity, and sleep–wake synchronized to the light–dark cycle (96, 97). As a result, these lead to having better physical, intellectual, and emotional performance (3, 94), as well as improved mental health (98). Our data emphasize that this line of complementary therapeutic approach seems essential in the treatment of SUD and even more so in SUD + MDD.

This study has some limitations. Our sample is comprised with non-randomized groups without a control group, and the suicide attempts were collected by a self-reported strategy and was retrospective but without recording the attempted method and seriousness; although we compared this information with medical registrations, we do not exclude that such data might be biased. It is the first study made in this line in Iran so our results can be not generalized to other mental disorders. Therefore, we suggest that future studies investigate more aspects of SUD and severe mental disorders comorbid to SUD according to their circadian functions, sleep characteristics, and QOL to achieve the optimum levels of recovery in treatment programs. Only the male gender was investigated, so it is suggested to consider also the female gender in future works. However, we provide for the first time new insights to previous research exploring circadian functioning and QOL in SUD patients with and without comorbid MDD. Other strong points were an accurate diagnosis of each group and an exhaustive evaluation of clinical characteristics and circadian functioning. Additionally, an appropriate number of patients in each group was included, especially in SUD + MDD compared with most of the previous research, employing a cross-sectional design which permitted us to ascertain the contribution of each psychiatric condition.

## Conclusion

This study has examined circadian functioning and QQL in SUD patients with and without comorbid MDD. The SUD + MDD group presented more concomitance of organic pathology, a higher amount of psychiatric disorders history, as well as more suicide attempts than the SUD group. Instead, SUD patients reported more law or legal problems and higher rate of opium, crystal, and heroin use compared with the SUD + MDD ones. Besides, the SUD + MDD patients showed worse scores in sleep quality, and with the total PSQI scores negatively related to the age of SUD onset and positively related to the severity of MDD, respectively. Since we did not find any interaction among circadian typologies and the groups with respect to the sleep quality, this reinforces the idea that the quality of sleep probably plays a more important role than the circadian typology for SUD and SUD + MDD patients. The few previous studies that have been done on dual disorders described more affectation than in SUD; our study adds evidence of more problematic lifestyles in SUD + MDD patients, even after controlling confounding factors. We found that SJL and severity of MDD had a negative relationship with physical health in the SUD + MDD group. Regarding sleep latency, SUD + MDD patients with the most latency reported the lowest physical health, while SUD patients with the highest sleep latency reported the lowest psychological health. The high prevalence of SP in our study, regardless of the group, highlighted the importance of a precise assessment of sleep disturbances in future studies about patients with SUD with/without depression comorbidity. Future research should consider our results with respect to the mentioned limitations for knowledge promotion in this scope and related outcomes to design better and more effective treatment processes, which could be cost effective and can improve the chance of success of the treatment protocol.

## Data Availability Statement

The raw data supporting the conclusions of this article will be made available by the authors, without undue reservation.

## Ethics Statement

The study involving human participants was reviewed and approved by the Ethics Committee of the Research Committee of the University of Barcelona (IRB00003099) and authorization from the research center of Shiraz University of Medical Science. All patients provided signed consent for study participation. The patients/participants provided their written informed consent to participate in this study.

## Author Contributions

AA conceptualized and designed the study. IH was responsible for the data acquisition and curation. IH and AA analyzed and interpreted the data and wrote the manuscript. JEM-A, KH, and JFN critically reviewed the manuscript text. All authors read and approved the final manuscript.

## Funding

This research was funded by the Spanish Ministry of Economy, Industry and Competitiveness (PSI2015-65026-MINECO/FEDER/UE), the Generalitat de Catalunya (2017SGR-748), the Spanish Ministry of Science and Innovation (PID2020-117767GB-I00/AEI/10.13039/501100011033), and Research Group CTS-195 (Junta de Andalucía, Spain). The funders had no role in the study design, data collection and analyses, decision to publish, or preparation of the manuscript.

## Conflict of Interest

The authors declare that the research was conducted in the absence of any commercial or financial relationships that could be construed as a potential conflict of interest.

## Publisher's Note

All claims expressed in this article are solely those of the authors and do not necessarily represent those of their affiliated organizations, or those of the publisher, the editors and the reviewers. Any product that may be evaluated in this article, or claim that may be made by its manufacturer, is not guaranteed or endorsed by the publisher.
